# VetCompanion

**DOI:** 10.5195/jmla.2017.119

**Published:** 2017-01

**Authors:** Laura L. Pavlech

VetCompanion is an evidence-based, point-of-care resource for small animal (canine and feline) veterinarians, veterinary students, and veterinary technicians. It is one of the few veterinary resources specifically designed for point-of-care use. VetCompanion was co-founded in 2014 by Matthew P. Mauer, a doctor of osteopathy who served as a medical director at the New York State Department of Health and currently works as a medical editor at Elsevier, and Jennifer Garcia, a board-certified veterinary internist, certified continuing medical education professional, medical writer, and editor [1]. Mauer serves as chief executive officer (CEO) and editor-in-chief of VetCompanion, while Garcia serves as chief veterinary medical officer. In their respective roles, Mauer reviews all content, and Garcia writes and reviews content [2]. Contract medical writers with doctor of veterinary medicine degrees also write for VetCompanion. The editorial board of VetCompanion consists of five veterinarians: two are board-certified internists at academic institutions, and the remaining three are general practitioners in private practice [1]. Clinicians at NorthStar VETS—a veterinary emergency, trauma, and specialty center in New Jersey—provide expert review as needed.

## OVERVIEW

Registration and payment for VetCompanion are straightforward and can be completed from the product website. Each user has a unique, password-protected account. Subscriptions that accommodate institutional credentials or Internet protocol (IP) address recognition were not offered at the time of this review (August 2016). VetCompanion is a web-based resource; no mobile app is currently available. The site does not provide options for downloading, emailing, or saving content for offline use, which may limit its utility in veterinary practices that lack wireless service or for ambulatory veterinarians with sporadic access to wireless service. No access problems were experienced on any of the devices (Macbook Pro, Dell Latitude, iPad Air, iPhone 5) or browsers (Mozilla Firefox, version 47.0; Google Chrome, version 52.02743.116; Safari, version 9.3.2 on iPad) on which the site was tested.

The VetCompanion interface is clean, intuitive, and mobile-responsive. Upon logging in to VetCompanion, users are presented with a home page that offers a search box as well as the ability to browse all topics alphabetically. A menu bar at the top of each page provides links to: Topics, which is an alphabetical list of all topics; Specialties, where topics are organized according to clinical specialty; Recently Viewed; News, which displays information about the site, such as new topics or updates to existing topics; and Suggestions, where users can submit suggestions about the site’s content or design. One topic, Conversions—which provides formulas for converting weight, temperature, and other measures—does not fit into the Specialties organization scheme, making it difficult to find. A dedicated link in the menu bar at the top of each page would make this topic easily accessible from any page.

## SEARCHING

Queries for diseases (diabetes, congestive heart failure, urinary tract infection); clinical signs (hematuria, epistaxis); and procedures (thoracocentesis) were used to test the functionality of VetCompanion’s search. According to VetCompanion’s CEO, the site’s search matches the words in a query to topic titles, summaries, and author- or editor-applied tags [2]. These tags are synonyms for or describe important elements of the topic. The search does not currently support Boolean operators (AND, OR, NOT), although this function might be added in the future. A test of phrase searching in VetCompanion was inconclusive. A query for “hemolytic anemia” returned some topics in which the phrase was present, for example, babesiosis, and others in which it was not, for example, aplastic anemia.

## CONTENT

Topics are selected for inclusion in VetCompanion based on consultation with practicing veterinarians and members of the editorial board; review of recent guidelines, consensus statements, and research; perusal of frequently asked questions on veterinary websites; and user recommendations [1]. A precise count of the number of topics covered in VetCompanion was difficult to obtain, because a single topic could be in more than 1 category. Overall, coverage appeared sparse and focused more on internal medicine (123 topics) than surgery (10 topics). Common surgical (or potentially surgical) emergencies, such as gastric dilatation volvulus and dystocia, were not covered. However, VetCompanion is a relatively new resource, and new topics are continually added.

**Figure 1:**
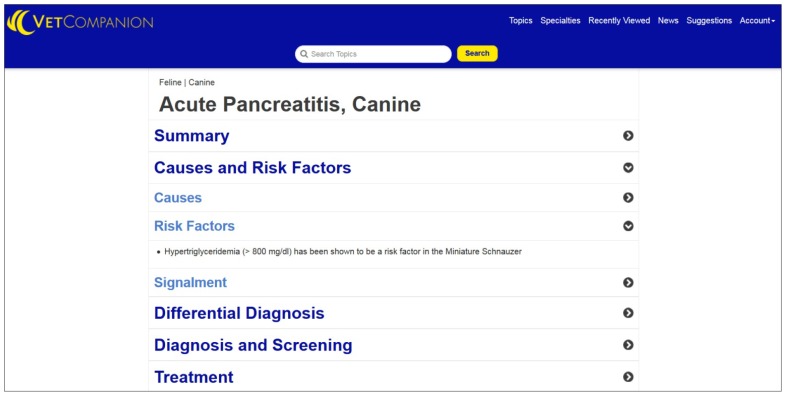
VetCompanion sample

The title of the topic and the species to which the topic is applicable (canine, feline, or both) appear at the top of each monograph, and date of last update appears at the bottom. Content is updated as new information becomes available. The authors of the monograph are not listed. Each monograph is arranged in a series of expandable topic page headings and subheadings: Summary, Causes and Risk Factors, Differential Diagnosis, Diagnosis and Screening, Prevention, Treatment, Follow-up, Prognosis, and Evidence (Figure 1).

This arrangement mirrors how a clinician works through a case, with a few exceptions. For example, the Diagnosis and Screening heading is positioned after the Differential Diagnosis heading, yet differential diagnosis typically takes place after initial evaluation of the patient. A more intuitive arrangement could be achieved by placing the subheadings Signs and History and Physical Exam under a separate heading called Clinical Presentation. This new heading should be positioned before the Diagnosis and Screening and Differential Diagnosis headings. Unfortunately, the monograph pages do not have a table of contents with links to each heading and subheading, which makes navigating to a particular section inefficient, particularly on a mobile device.

Information in VetCompanion is presented in brief, bulleted sentences. After reviewing several monographs, this reviewer felt that the content was not detailed enough to be clinically useful. Also, the monographs examined for this review had a few omissions and unusual recommendations. For example, the monograph for congestive heart failure did not mention echocardiography for diagnostic follow-up. In the pancreatitis monograph, methadone was one of the drugs recommended for pain control. While a valid recommendation, methadone is rarely used in small animal practice in the United States.

Drug dose recommendations, culled from multiple sources, are provided in the topic monographs. VetCompanion’s terms of use encourage users to consult product information sheets and “an appropriate drug information database” [1] to confirm drug use and dose information. Currently, drugs do not have their own monographs and, therefore, are not searchable in VetCompanion. In August 2016, VetCompanion announced that the current edition of *Saunders Handbook of Veterinary Drugs* would be added to the site [2]. Initially, the electronic version of the book will be accessible via a link from the home page. Later, content from the book will be integrated into the VetCompanion site, so that each drug becomes its own searchable topic. This reliable, widely used resource will be a valuable addition to the site.

Few monographs include images, and the images that are included are not always useful. For example, the feline diabetes monograph has an image of a glucometer, syringe, and pill bottle. Fortunately, for topics where a visual component is particularly useful, such as dermatological conditions, images are provided.

## FEATURES FOR EVIDENCE-BASED VETERINARY MEDICINE

Specific studies are mentioned in the text of some monographs. Unfortunately, in-text citations are not included, so the user cannot connect the information in the monograph to the references in the Evidence section. In many monographs, studies are not mentioned at all. For example, the pancreatitis monograph states that canine pancreatic specific lipase (cPLI), a blood test that can aid in the diagnosis of pancreatitis, has a high sensitivity and specificity but does not include either the percentages or the reference for this information. If VetCompanion is truly designed to help clinicians make evidence-based decisions, then more detailed information and in-text citations with links to the full reference in the Evidence section need to be included.

References in the Evidence section are organized under subheadings: Guidelines and Consensus Statements, Systematic Reviews/Meta-Analyses, Randomized Controlled Trials, Other Studies or Reviews, and Additional Readings. According to VetCompanion’s website, “All pertinent guidelines, consensus statements, systematic reviews, meta-analyses, randomized, controlled trials, and observational studies are included for each clinical topic” [1]. Sources reviewed for topic selection and evidence include databases, such as PubMed and VetSRev, and websites of professional organizations, such as the American College of Veterinary Internal Medicine, American Animal Hospital Association, and British Small Animal Veterinary Association. Textbooks are also utilized when preparing monographs, and other veterinary professional websites are consulted as needed. PubMed is the only bibliographic database listed on the VetCompanion website as a source searched for evidence, yet databases such as Scopus and CAB Abstracts have been shown to have more extensive coverage of veterinary journals [3]. Inclusion of another bibliographic database on the list of VetCompanion’s sources would enhance the user’s confidence that the veterinary literature had been thoroughly searched for evidence.

Using a system derived from the Strength of Recommendation Taxonomy (SORT), VetCompanion assigns a level 1, 2, or 3 to each study and A, B, or C to each guideline or consensus statement in the Evidence section. The levels of evidence (LOE) criteria are described on the Methodology page of VetCompanion’s website. This information is not available within the resource itself, either as a link from the menu bar or adjacent to the reference. The link to the abstract, located at the end of many references in the Evidence section, is equally problematic. Links to abstracts are not provided for all references, and, for journal articles, the destination of the links varies, with some going to the PubMed record and others to the article on the journal’s website. According to VetCompanion’s CEO, an editorial decision was made to only provide links to abstracts for guidelines, consensus statements, and studies with an evidence level of 1 or 2 [2]. He also noted that variations in the destination of the links were unintentional. Not providing a readily accessible explanation of either the LOE criteria or the links to abstracts diminishes the value of VetCompanion as a tool for evidence-based veterinary medicine.

## COMPARISON TO OTHER RESOURCES

For many years, Veterinary Information Network (VIN), a professional networking and continuing education site, was one of the few online resources for veterinarians. VIN does not offer evidence-based summaries of diseases and is not designed, nor was it intended to be used, as a point-of-care resource. Therefore, despite its longevity and popularity among veterinarians, it cannot be considered a direct competitor of VetCompanion.

In 1996, Vetstream, a British company offering digital solutions for veterinarians, launched Vetlexicon, a peer-reviewed, point-of care resource for veterinarians. This resource consists of four separate “services” or databases: Canis (canine), Felis (feline), Lapis (rabbits), and Equis (equine). A fifth service for cattle, Bovis, will be launched at the end of 2017. Compared to VetCompanion, Vetlexicon covers more species and topics, provides a broader array of content, and has a more extensive list of contributors and reviewers. For example, Canis covers 1,035 diseases; 362 diagnostic and surgical techniques; 543 formulary items; and has 4,359 images, videos, and sounds; 175 owner factsheets; and over 500 contributors and reviewers from around the world [4]. Content in Vetlexicon is linked to articles in VetMed Resource, which provides records from CAB Abstracts and PubMed. Unlike VetCompanion, Vetlexicon allows advertising in its services. An annual subscription to a single Vetlexicon service costs more than an annual subscription to VetCompanion.

This reviewer was left with the impression that VetCompanion is a work-in-progress without a well-defined audience. The small number of board-certified contributors and reviewers, narrow scope of content, and inconsistency with how evidence is presented limit the usefulness of the current iteration of VetCompanion for veterinary students, technicians and clinicians, and librarians who support these populations.
